# The impact of surgery and mild hyperthermia on tumor response and angioneogenesis of malignant melanoma in a rat perfusion model

**DOI:** 10.1186/1471-2407-4-53

**Published:** 2004-08-23

**Authors:** Joerg Pelz, Marco Mollwitz, Christian Stremmel, Jonas Goehl, Arno Dimmler, Werner Hohenberger, Thomas Meyer

**Affiliations:** 1 Department of Surgery, University of Erlangen-Nuremberg, Germany; 2 Institute of Pathology, University of Erlangen-Nuremberg, Germany

**Keywords:** melanoma, angioneogenesis, isolated limb perfusion, hyperthermia

## Abstract

**Background:**

The aim of this experimental study was to determine the effect of mild hyperthermia on tumor response and angioneogenesis in an isolated limb perfusion model with a human melanoma xenograft.

**Methods:**

A human melanoma xenograft was implanted into the hindlimbs of 30 athymic nude rats. The animals were randomized into five groups: group I: control, group II: sham group, group III: external hyperthermia with a tissue temperature of 41.5°C for 30 minutes without ILP, group IV: normothermic ILP (tissue temperature 37°C for 30 minutes, group V: hyperthermic ILP (tissue temperature 41.5°C for 30 minutes). Tumor response was evaluated by tumor size determination and immunohistochemical analysis 6 weeks postoperatively. Tissue sections were investigated for expression of CD34 and basic fibroblast growth factor (bFGF).

**Results:**

Average tumor volumes of the controls (I) increased from 105 mm^3 ^to 1388 mm^3^. In the sham operated group (II) tumor volumes were significantly larger than in group I. Tumor volumes in group IV were significantly smaller than in group I and lowest in group V. There were no significant differences in size between group I and group III after six weeks. In group III and IV each, 5 animals showed tumor progression and one had a partial tumor response. In group V only 2 animals showed tumor progression. Immunhistochemical analysis of the tissue sections demonstrated that angioneogenesis was more pronounced in group II than in group I and less pronounced in group IV and V compared with group I.

**Conclusions:**

Our results suggest that even a surgical manipulation such as a skin incision promotes tumor growth, probably by induction of growth factors like bFGF.

External hyperthermia of 41.5°C tissue temperature for 30 minutes only has no impact on tumor growth and angioneogenesis in vivo.

## Background

Hyperthermic isolated limb perfusion is an established treatment for multiple, locoregional intransit metastases of malignant melanoma and soft tissue sarcomas of the extremities. It was introduced 1957 by Creech and Krementz [1]. The procedure allows a high locoregional concentration of cytostatics with few systemic side effects [2]. Isolated limb perfusion achieves local tumor control in a high percentage of cases. Complication rate of perfusion is acceptably low. A surviving model for isolated perfused rat hindlimbs was reported by Wu et al. [3] based on the perfusion model of Nagel et al.[4].

Angioneogenesis has been recognized for many years to play a central role in the growth of primary tumors and the formation of metastases in general [5,6]. Melanomas express basic fibroblast growth factor (bFGF) and fibroblast growth factor receptor-1 (FGFR-1) in their dermal nevocytes and in the stroma. bFGF promotes angiogenesis *in vivo *[7] and *in vitro *[8]. Antisense targeting of bFGF/FGFR-1 in malignant melanomas blocks intratumoral angioneogenesis [9].

Hyperthermia inhibits angioneogenesis. Some of the antineoplastic effects of hyperthermia are caused by ischemia due to obstruction or destruction of the tumor vessels. Eikesdal et al. described by hyperthermic temperatures disrupture of 25–50 % of the vasculature in malignant tumors. The heated tumors (44°C) had a blood flow reduction of 40–60 % after 24 hours [10].

*In vitro *data indicate that capillary endothelials cells of malignant neoplasms are more thermosensitive than endothelial cells of normal tissues. The vascularization of tumors can be significantly damaged at temperatures which may alter but do not damage the vasculature of normal tissue [11]. The extent of the proliferation of vessels is also inversely related to temperature [12]. The critical temperature at which a direct vascular damage occurs is between 42.7 and 43.7 degrees C [13].

Hyperthermia increases the effects of angioneogenesis inhibitors on tumor growth and tumor angioneogenesis [14]. The aim of this experimental study was to determine the impact of hyperthermia on tumor response and angioneogenesis in an isolated limb perfusion model.

## Methods

Thirty inbred male nude athymic rats (Rowett rnu/rnu) weighting 200 to 280 g were used in this study. Animals were kept separately during the experiment with 12 hours of light per day. They were fed a standard laboratory diet and tap water *ad libidum*. Maintenance and care of all experimental animals were carried out according to the guidelines of the local responsible Animal Protection Commission and carried out in compliance with national guidelines (National Institute of Health for Use of Laboratory Animals).

A solid sample (2 × 2 × 2 mm) of a human melanoma xenograft (SK-MEL-3), derived from a lymph node melanoma metastasis, was implanted into the hindlimbs of 30 nude rats. The largest width and the maximum tumor diameter perpendicular to the width were measured with a micrometer. Only tumors with a diameter greater than > 12 mm in the largest diameter were included in the study.

The details of the perfusion system have been described previously [4]. The equipment consisted of a miniature oxygenator, a heat exchanger and a roller pump. Venous blood of the limb was oxygenated in the oxygenator (99.15% O_2_; 0.85% CO_2_) and warmed in the heat exchanger. The warmed arterialized perfusate is driven by a roller pump with two synchronously running pump-heads on a single axis for the arterial and venous lines (Masterflex^®^). The outer diameters of the tubes corresponded with the diameter of the femoral vessels of nude rats weighting about 250 g (arterial 0.7 mm; venous 1.0 mm).

Rat hindlimbs were perfused with ringer's solution and sodium heparin (25 IU/100 μl saline) at 37 or 41.5 degrees C for 30 minutes with a flow rate of 4 ml/min. External hyperthermia was applied by an infrared lamp positioned in a fixed distance to the tumor.

For temperature measurement during limb perfusion, a nickel-chrom-nickel thermocouple of 0.6 mm in diameter (Standard Integrated Thermocouple Thermocoax, Phillips, Hamburg, Germany) was placed at the macroscopic tumor margin. The thermocouple was calibrated before use in a high-precision water bath. Baseline temperature was recorded for 5 minutes before treatment. Temperature was continuously measured during application. In group I (control) and group II (sham operated) temperature measurement was not performed.

The animals were randomized into a control and four study groups of six animals each: group I: control (no therapy), group II: sham group (skin incision without ILP), group III: external hyperthermia with a tissue temperature of 41.5°C for 30 minutes without ILP, group IV: normothermic ILP (Ringer's solution as perfusate, tissue temperature 37°C for 30 minutes, group V: hyperthermic ILP (tissue temperature 41.5°C for 30 minutes). ILP was subsequently performed with the above-mentioned parameters. Antineoplastic agents were not applied.

**Figure 1:**
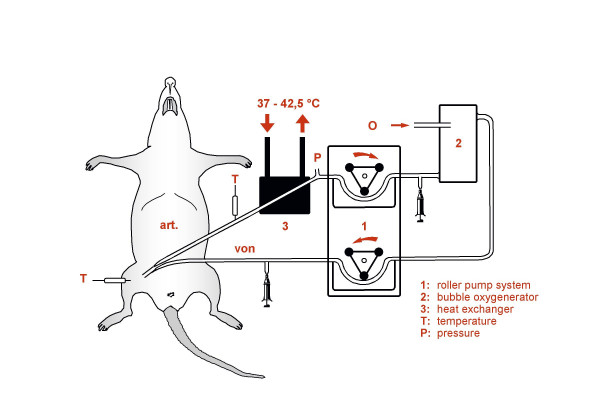
Diagrammatic outline of the experimental setup. 1: roller pump system; 2: bubble oxygenerator; 3: heat exchanger, T: temperature

The rats were anaesthetized with Ketamin (Ketanest^®^, 80 mg/kg i.m.; Pearl-Davis, Berlin, Germany) and Xylazin (Rompun^®^, 10 mg/kg i.m.; Bayer, Leverkusen, Germany), and fixed in a supine position. For perfusion, the animals underwent a 3-cm incision of the groin. The left femoral artery and vein were isolated using an operation microscope. A vascular clip was placed across the artery and a silicon catheter (outer diameter 0.7 mm) was introduced via a transverse arteriotomy. The femoral vein was cannulated with a silicon catheter of 1 mm outer diameter (Fig. 1). Tourniquets were applied around the left hindlimb to ensure isolation. Rat hindlimbs were perfused at a flow rate of 4 ml/h, for 30 min. After treatment, the silicon catheters were removed, and the vein and the artery were sutured (8/0 Prolene), to restitute normal blood flow. After the isolated limb perfusion the wound was closed in two layers. Total operation time rated about 90 min.

During the first 5 days after operation the rats were weighed daily, and once a week there-after. The animals were kept under standardized conditions and were killed by an overdose of anesthetic and cervical dislocation 6 weeks after treatment. The greatest tumor width and the maximum diameter perpendicular to the width were macroscopically measured with a micrometer. Lesion volume was calculated using the formula v = 4 × π × a × b 2/3, in which a and b are the radii of the measured axes. The tumor was then completely sectioned. Each tumor was divided into two parts. One part was fixed in 4 per cent formalin solution for 5 days, embedded in paraffin and stained with hematoxylin and eosin (H&E). The other part was fixed in 0.9 % saline solution, frozen and stored at -80°C until use.

Vascular density and tumor morphology were evaluated by immunohistochemistry. Tumor tissue was tested for expression of basic fibroblast growth factor (bFGF) and CD34.

Tissue specimens were fixed in 10 % formaldehyde and embedded in paraffin according to routine protocols. Sections 4 μm thick were deparaffinized and rehydrated using graded ethanols following routine protocols. For antigen retrieval all sections were microwave treated for 20 minutes in target retrieval solution (for bFGF; DAKO, Glostrup, Denmark) or 10 mM citrate buffer (for CD34) at 700 W. The sections were incubated overnight with primary mouse anti-bFGF (1:50; Becton Dickinson, Heidelberg, Germany) and anti-CD34 antibody (1:100; Immunotech, Hamburg, Germany). After rinsing in Tris buffer detection was carried out with a biotinylated secondary rabbit anti-mouse antibody diluted 1:50 (DAKO, Glostrup, Denmark) for 30 minutes at room temperature. The sections were washed again as above in Tris buffer and then incubated with streptavidin-biotin-alkaline-phosphatase (10 μl each solution A+B (Sigma, Deisenhofen, Germany) in 10 ml Tris). Immunohistochemical staining was visualized with Fast Red (2 mg Naphtol-As-Mx-phosphate, 10 μl 1 M Levamisol and 10 mg Fast-Red (Sigma) in 0.2 ml N, N-dimethylformamide, (Merck, Darmstadt, Germany) and 9.8 ml 0.1 M Tris-HCl buffer at pH8.6). The sections were rinsed in H_2_0 and counterstained with hemalaun. Negative controls without primary antibody were run for each sample.

Tumor response was graded according to the system described by de Wilt et al. [15]. Progressive disease (PD) was defined as a tumor size > 125 % of the size at the time of treatment, in tumor sizes of 100 ± 25 % no change (NC) was stated. Response was graded as partial (PR) if tumor sizes was between 10 and 75 % and complete (CR) for tumor size < 10 % of its original diameter.

In areas of intense neovascularized spots microvessels were counted in a 100× field. A microvessel was defined as a lumen surrounded by a rim of endothelial cells highlighted by immunostaining with anti-CD34 antibodys or anti-bFGF antibodys. Five separate intense neovascularized areas were assessed, and the mean was calculated as microvessel density of each tumor. The score was assessed by two independent observers.

Data were analyzed using SPSS/PC+ statistical software. The mean and range of tumor and lesion volumes were calculated for each group. For comparison of tumor volume and microvessel density between the diffent groups a non-parametric test was used (Kruskal-Wallis). A p value < 0.05 was considered to be significant.

## Results

During intervention, the required limb tissue temperature was reached within 8–12 minutes in group IV and 10–15 minutes in group III. Stable temperatures were then maintained for a further 30 minutes with a mean of 41.4°C ± 0.5. To reach limb tissue temperature the perfusion fluid maintained 43°C ± 0.4.

### Tumor and lesion Volumes

**Figure 2:**
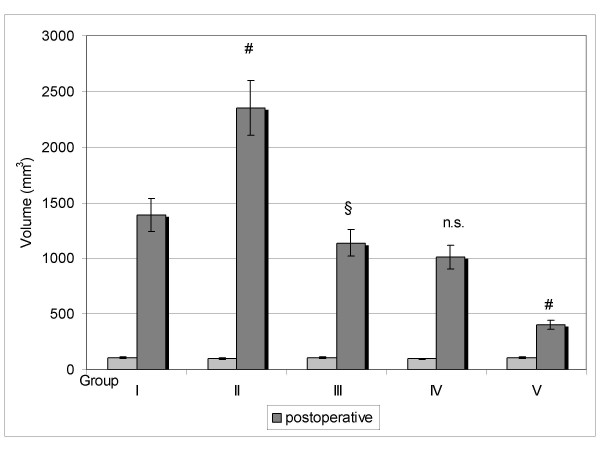
The mean tumor volumes + SEM of groups I to V (n = 30) 6 weeks after treatment; Group II, III, IV and V compared to group I (§: *P *= 0.036 ; #: *P *= 0.021 (Kruskall-Wallis) n.s.:not significant).

Before treatment, the mean (s.e.m) volume of the treated tumors in groups I, II, III, IV and V was 105 mm^3 ^(4.3), 98 mm^3 ^(5.7), 108 mm^3 ^(3.4), 95 mm^3 ^(5.0) and 107 (4.1) mm^3 ^respectively, with no significant difference between the groups. Average tumor volume of the controls (I) increased to 1388 mm^3 ^(101) during six weeks. In the sham operated group (II) tumor volume was significantly larger than in group I (2350 mm^3 ^(198.6), *P *= 0.021). Tumor volume in group IV was significantly smaller (1009 mm^3 ^(122.5)) than in group I and lowest in group V 405 mm^3 ^(103.6) (*P*= 0.036 and *P*= 0.021, respectively.). There were no significant differences in size between group I and group III (1135 mm^3 ^(99)) after six weeks (*P *> 0.05)(Fig. 2).

### Body weight

After the perfusion, body weight decreased in all groups during the first 5 days with a maximum of 8 %. In the following weeks, body weight mounted up to 130 % of the preoperative value. In the last week of the experiment, the weight almost reached a plateau. No significant differences between the five experimental groups were found.

### Histology

In the H&E staining the untreated melanoma (group I) presented as a subcutanous unilocular nodule of moderate differentiation. The tumor showed mainly tubular structures with vacuole like lumen formation and was infiltrated with fibrous septa. The spontaneous rate of necrosis was estimated to be 20 %. Numerous mitoses were detected. Tumor morphology did not change with ageing or tumor size. Histology was similar in group II.

In group V the tumors showed signs of irreversible cell damage after treatment. Tumor cells displayed clear shrinkage and partial loss of cell contact. Thromboses of the larger adjacent vessels were found on the tumor-skin border. Macrophage infiltration was present in all groups, but was more numerous pronounced in group V. In group III and IV there were less signs of irreversible cell damage after treatment than in group V.

**Figure 3:**
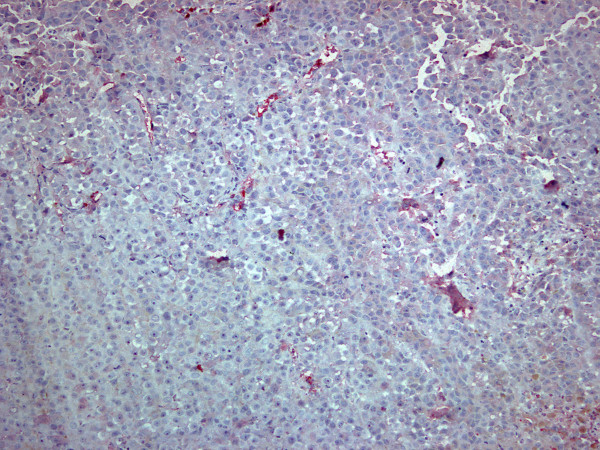
Immunohistochemical staining for bFGF in group I (control): Microvessels are represented by red clusters, which stand out sharply from other tissues.

**Figure 4:**
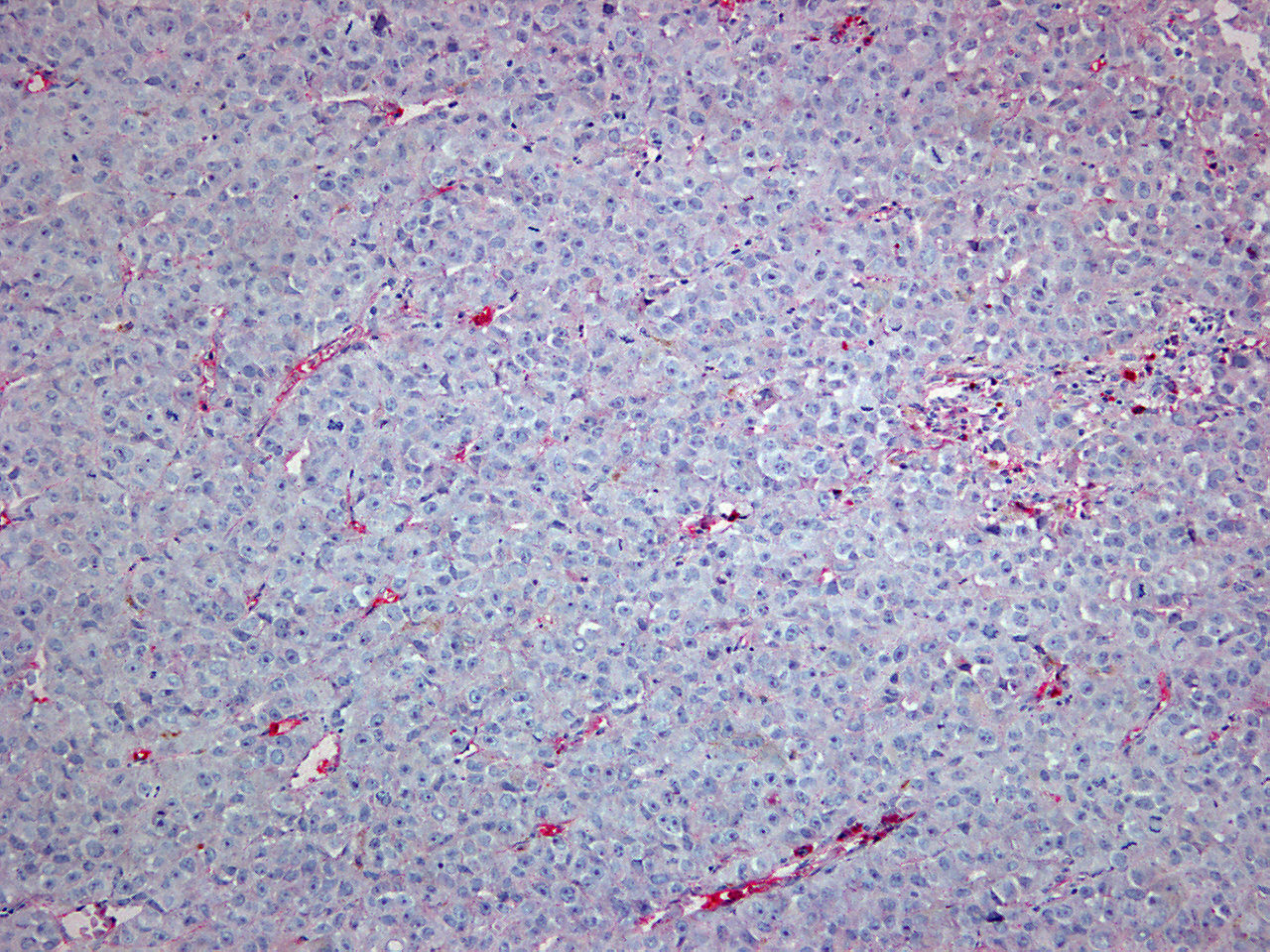
Immunohistochemical staining for bFGF in group II (sham operated): More microvessels are represented in the margin of the tumor.

**Figure 5:**
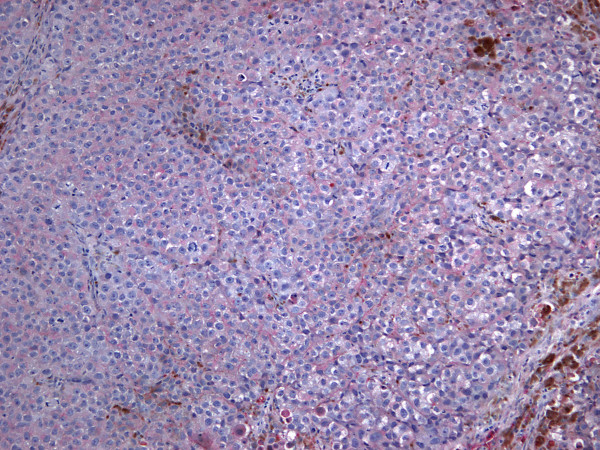
Immunohistochemical staining for bFGF in group V (HILP): Angioneogenesis is less pronounced as in group I. Brown clusters represent melanin.

Immunhistochemical analysis of the histological sections for bFGF demonstrated that microvessel density of group I (Fig 3) ranged from 18 to 23 with a mean value of 20. Angioneogenesis was more pronounced in group II (Fig 4) than in group I (p = 0.003) and less pronounced in group IV and V (Fig 5) compared with group I (p = 0.023, p = 0.001). There were no significant differences in the expression of angiogenetic markers between group I and group III (p >0.05).

Immunhistochemical analysis of the histological sections for CD34 showed that microvessel density of group I ranged from 17 to 23 with a mean value of 19. Angioneogenesis was more pronounced in group II than in group I (p = 0,001) and less pronounced in group IV and V compared with group I (p = 0.023, p = 0.017). There were no significant differences in the expression of angiogenetic markers between group I and group III (p > 0.05).

Strong expression of bFGF and CD34 was found in the cytoplasm of intimal endothelial and medial smooth muscle cells. Expression of bFGF and CD34 was stronger at the invasion front than in the centre of tumors.

Summarized data of the expression intensity of bFGF and CD34 in the tumors are given in table 1.

### Tumor remission

In group I and II tumor progression was observed in all animals macro- and microscopically. In group III and IV 5 animals showed tumor progression and one had a partial tumor response. In group V, 2 animals showed tumor progression, 2 had a complete remission. One had a partial tumor response and one had no change in tumor response (table 2).

## Discussion

A phenomenon that has been described by numerous authors is the rapid increase of tumor growth following surgical manipulation [16,17].

This was also observed in our sham-operated group of animals (group II) in which tumors grew more rapidly than in the control group (group I). This implies that resection is associated with an accelerated growth in residual tumors. It is assumed that the surgical manipulation induces liberation of growth factors that in addition to their effect on healing also have a stimulating effect on tumor proliferation.

This hypothesis is supported by the results of our immunohistochemical investigations, which revealed an enhanced expression of fibroblast growth factor (bFGF) in group II. In an intraperitoneal tumor model, EGGERMONT et al. showed that the surgical intervention of laparotomy induces an increase of carcinomatosis in the entire peritoneal cavity. At the same time, the immunotherapeutic effect of interleukin 2 and natural killer cells is significantly reduced. It must therefore be speculated that surgical trauma induces a temporary immunosuppressive effect that influences tumor growth [16,18,19].

In our experiments, group V (hyperthermic perfusion) showed a significant slowing of tumor growth in comparison with the control group. This was also confirmed by the immunohistochemical investigations that showed a reduction in the expression of relevant markers of the vascular endothelium. Indeed, two of the six animals occurred a complete remission of their tumor. The observation that two animals in this group experienced tumor progression can probably be explained by the duration of the intervention, since factors affecting the kinetics of cell death during hyperthermia are not only the maximum temperature induced, but also the duration of the elevated temperature.

In an overview report BHUYAN noted that, as a function of various tumor cell types, a temperature of 43°C must be applied between 30 and 150 minutes to irreversibly damage the tumor cells. At a temperature of 45°C, the application time required varied between 13 and 85 minutes [20]. In our experiments, the application time was only 30 minutes at a temperature of 41.5°C. Nevertheless, the relatively short application time also had an appreciable damaging effect on the metastatic vessels.

Although the temperature in group III (external hyperthermia) was also 41.5°C applied for 30 minutes, no significant changes in the growth pattern of the tumor could be observed. Furthermore, the histological and immunohistochemical evaluations showed a mild hyperthermic damage at best. A comparison of treatment groups III and V revealed no differences in terms of duration of treatment and induced tissue temperatures.

In contrast to group III, however, hyperthermia in group V has been mediated directly by the vascular system. In normal tissues subjected to warming, blood perfusion increases reactively via dilatation of the vessels together with an increase in vessel wall permeability. A decisive factor is that the tumor cells themselves, as well as the endothelial cells of tumor vessels are more thermosensitive than normal tissue [16,21,22]. Other authors have also shown that hyperthermia is capable of destroying vascular architecture, so that perfusion of the tumor is reduced. The sensitivity of various tumors, however, varies considerably [14]. ILP and other vasculotoxic effects like TNFalpha in combination with melphalan or doxorubicin increase the uptake of these drugs of three to six times [23].

Heat transfer between the vascular system and the tissue is determined in particular by the nature of the blood vessels. Important factors are the number, length, and diameter of the vessels, and also the velocity of blood flow [24]. CREZEE described the relationship between tumor perfusion and heating of the tumor tissue under systemic hyperthermia. He could show that the induction of heat in the tumor depends on the density of the blood vessels within the tumor itself. The more vessels in the tumor exist, the easier it is to heat the lesion via the vascular system. Heattransfer is particularly pronounced with large-calibre blood vessels or high flow velocity, and its rate is all the greater the higher the temperature gradient between the tumor tissue and the blood [25,26].

On the other hand, tumor vasculature carries away the externally applied heat (group III) which is then no longer available to destroy the tumor cells. In this case, the temperature gradient decreases from the tumor to the vascular system.

Furthermore, in the presence of hyperthermia, microcirculation in the tumor tissue is more affected than in normal tissue [27]. In addition, manipulation to the vascular system, and thus oxygenation of the tumor, also appears to play an important role. Although the body's own oxygenated blood is added to the perfusate, the dilution effect together with the clamping time, leads to a relative reduction in perfusion. It is known that hypoxia, in conjunction with a decrease in the pH and energy status of the cell, enhances thermosensitivity [28-30], and this increase correlates with the size of the tumor [31].

Our results have also confirmed that surgical manipulation of the vascular system and perfusion have an influence on the tumor. Overall, the effect of the intravascular application of hyperthermia appears to be mediated by a combination of hypoxia and heat.

## Conclusions

Our results suggest that even a surgical manipulation such as a skin incision promotes tumor growth, probably by induction of growth factors like bFGF. External hyperthermia of 41.5°C tissue temperature for 30 minutes has no impact on tumor growth and angioneogenesis in vivo. Hyperthermic isolated limb perfusion effectively suppresses tumor angioneogenesis respectively tumor growth.

## Competing interests

None declared.

## Authors' contributions

JP carried out the treatment and drafted the manuscript.

MM carried out the treatment.

CS carried out the immunohistochemical studies.

JG participated in the design of the study.

AD carried out the histological studies.

WH participiated in the design of the study.

TM participiated in the design and coordination of the study.

All authors read and approved the final manuscript.

## Pre-publication history

The pre-publication history for this paper can be accessed here:



## Figures and Tables

**Table 1:** Summarized data of the expression intensity of bFGF and CD34

	CD34	P to group I	bFGF	P to group I
group I	19	----	20	----
group II	28	0.001	26	0.003
group III	20	> 0.05	21	> 0.05
group IV	27	0.023	26	0.023
group V	16	0.017	16	0.001

**Table 2:** Tumor response to treatment of isolated limb perfusion. Tumor diameters were measured and expressed as a percentage of the size at the time of treatment.

**response**	**group I**	**group II**	**group III**	**group IV**	**group V**
complete	0/6	0/6	0/6	0/6	2/6
partial	0/6	0/6	1/6	1/6	1/6
no change	0/6	0/6	0/6	0/6	1/6
progressive	6/6	6/6	5/6	5/6	2/6
